# Improving measurement-based care implementation in youth mental health through organizational leadership and climate: a mechanistic analysis within a randomized trial

**DOI:** 10.1186/s13012-024-01356-w

**Published:** 2024-03-28

**Authors:** Nathaniel J. Williams, Mark G. Ehrhart, Gregory A. Aarons, Susan Esp, Marisa Sklar, Kristine Carandang, Nallely R. Vega, Lauren Brookman-Frazee, Steven C. Marcus

**Affiliations:** 1 Institute for the Study of Behavioral Health and Addiction, Boise State University, Boise, ID USA; 2 School of Social Work, Boise State University, Boise, ID 83725 USA; 3 Department of Psychology, University of Central Florida, Orlando, FL USA; 4 Department of Psychiatry, University of California, San Diego, CA USA; 5 University of Wisconsin-River Falls, River Falls, WI USA; 6 School of Social Policy and Practice, University of Pennsylvania, Philadelphia, PA USA

**Keywords:** Implementation leadership, Implementation climate, LOCI, Mechanism, Mediator, Measurement-based care, Youth, WISDOM

## Abstract

**Background:**

Theory and correlational research indicate organizational leadership and climate are important for successful implementation of evidence-based practices (EBPs) in healthcare settings; however, experimental evidence is lacking. We addressed this gap using data from the WISDOM (Working to Implement and Sustain Digital Outcome Measures) hybrid type III effectiveness-implementation trial. Primary outcomes from WISDOM indicated the Leadership and Organizational Change for Implementation (LOCI) strategy improved fidelity to measurement-based care (MBC) in youth mental health services. In this study, we tested LOCI’s hypothesized mechanisms of change, namely: (1) LOCI will improve implementation and transformational leadership, which in turn will (2) mediate LOCI’s effect on implementation climate, which in turn will (3) mediate LOCI’s effect on MBC fidelity.

**Methods:**

Twenty-one outpatient mental health clinics serving youth were randomly assigned to LOCI plus MBC training and technical assistance or MBC training and technical assistance only. Clinicians rated their leaders’ implementation leadership, transformational leadership, and clinic implementation climate for MBC at five time points (baseline, 4-, 8-, 12-, and 18-months post-baseline). MBC fidelity was assessed using electronic metadata for youth outpatients who initiated treatment in the 12 months following MBC training. Hypotheses were tested using longitudinal mixed-effects models and multilevel mediation analyses.

**Results:**

LOCI significantly improved implementation leadership and implementation climate from baseline to follow-up at 4-, 8-, 12-, and 18-month post-baseline (all *p*s < .01), producing large effects (range of *ds* = 0.76 to 1.34). LOCI’s effects on transformational leadership were small at 4 months (*d* = 0.31, *p* = .019) and nonsignificant thereafter (*p*s > .05). LOCI’s improvement of clinic implementation climate from baseline to 12 months was mediated by improvement in implementation leadership from baseline to 4 months (proportion mediated [*p*_*m*_] = 0.82, *p* = .004). Transformational leadership did not mediate LOCI’s effect on implementation climate (*p* = 0.136). Improvement in clinic implementation climate from baseline to 12 months mediated LOCI’s effect on MBC fidelity during the same period (*p*_*m*_ = 0.71, *p* = .045).

**Conclusions:**

LOCI improved MBC fidelity in youth mental health services by improving clinic implementation climate, which was itself improved by increased implementation leadership. Fidelity to EBPs in healthcare settings can be improved by developing organizational leaders and strong implementation climates.

**Trial registration:**

ClinicalTrials.gov identifier: NCT04096274. Registered September 18, 2019.

**Supplementary Information:**

The online version contains supplementary material available at 10.1186/s13012-024-01356-w.

Contributions to the literature

- This is the first study to experimentally test whether increases in organizational leaders’ use of implementation leadership and transformational leadership behavior contribute to improved organizational implementation climate which in turn contributes to improved clinician fidelity to a clinical intervention.
- We found that a leader-focused implementation strategy (a) improved implementation climate by increasing leaders’ use of implementation leadership and (b) improved clinician fidelity to a clinical intervention by enhancing implementation climate.
- This study offers robust evidence that organizational leaders can improve evidence-based practice implementation in healthcare settings by exhibiting implementation leadership behaviors and creating supportive implementation climates within their organizations.
- This study provides an example of rigorous multilevel mediation analyses for testing proposed mechanisms of implementation strategies.

## Background

Many implementation theories and frameworks in healthcare assert the importance of organizational leadership and organizational implementation climate for achieving high fidelity to newly implemented clinical interventions [1–6]; however, the evidentiary basis for these claims is thin. The usual standard for making causal claims in the medical and social sciences is demonstration of effect within a randomized controlled trial [7]; yet, recent reviews indicate no trials have tested whether experimentally induced change in organizational leadership or organizational implementation climate contributes to improved implementation of clinical interventions in healthcare [8, 9]. In the present study, we address this gap using data from the WISDOM (Working to Implement and Sustain Digital Outcome Measures) hybrid type III effectiveness-implementation trial. The WISDOM trial showed that a strategy called Leadership and Organizational Change for Implementation (LOCI) [10, 11], which targets organizational leadership and organizational implementation climate, improved fidelity to measurement-based care (MBC) in outpatient mental health clinics serving youth [12]. In this paper, we tested LOCI’s hypothesized mechanisms of change. Specifically, we tested whether improvement in clinic leadership contributed to improvement in clinic implementation climate and whether improved implementation climate in turn contributed to improved MBC fidelity.

### Measurement-based care in youth mental health

Measurement-based care is an evidence-based practice (EBP) that involves the collection of standardized symptom rating scales from patients prior to each treatment session and use of the results to guide treatment decisions [13]. Meta-analyses of over 30 randomized controlled trials indicate feedback from MBC improves the outcomes of mental health treatment relative to services as usual across patient ages, diagnoses, and intervention modalities [14–17]. There is also evidence MBC improves mental health medication adherence [18], reduces risk of treatment dropout [14], and is particularly effective for youths and for patients who are most at risk for treatment failure [14–16, 18].

Unfortunately, MBC is rarely used in practice. Only 14% of clinicians who deliver mental health services to youth in the USA use any form of MBC [19], and MBC usage rates are similarly low in other countries [20, 21]. When MBC use is mandated, less than half of clinicians view feedback and use it to guide treatment [22–24]. Digital MBC systems (i.e., measurement feedback systems) remove many practical barriers to MBC implementation by collecting measures from patients electronically (e.g., via tablet or phone) and instantaneously generating feedback [25]. However, even when these systems are available, clinician fidelity to MBC—defined as administering measures, viewing feedback reports, and using the information to guide treatment—is often substandard [22, 26]. Qualitative and quantitative studies of MBC implementation indicate clinicians’ work environments explain much of the variation in their attitudes toward, and use of, MBC [19, 27, 28], with organizational leadership and supportive organizational culture or organizational implementation climate identified as key determinants [13, 28–30].

### Mechanisms of the LOCI strategy

LOCI is a multicomponent organizational implementation strategy that engages organizational executives and first-level leaders (i.e., those who administratively supervise clinicians) to build an organizational climate to support the implementation of a focal EBP with fidelity [10, 31]. It includes two overarching components: (1) monthly organizational strategy meetings between executives and LOCI consultants/trainers to develop and embed policies, procedures, and practices that support implementation of a focal EBP and (2) training and coaching for first-level leaders, to develop their skills in leading implementation. Organizational survey data and feedback guide planning, goal specification, and progress monitoring for both components. The aim of these components is to develop an organizational implementation climate [32, 33] in which clinicians perceive that use of a specific EBP with high fidelity is expected, supported, and rewarded [34].

**Fig. 1:**
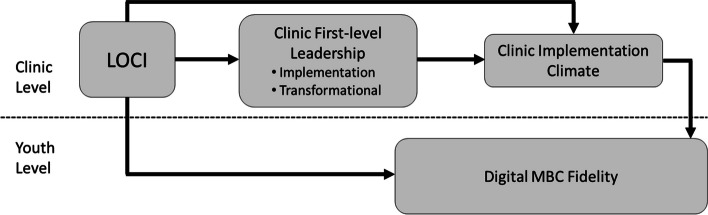
Study theoretical model. Note: *LOCI*, Leadership and Organizational Change for Implementation strategy; *MBC*, measurement-based care. First-level leaders are those who administratively supervise clinicians (e.g., clinical managers). Random assignment of clinics to LOCI (vs. training and technical assistance only) is expected to cause improvement in clinic-level implementation leadership, transformational leadership, and implementation climate for digital MBC (Aim 1). Improvement in implementation leadership and transformational leadership is expected to mediate LOCI’s effect on improved clinic implementation climate (Aim 2). Improvement in clinic implementation climate is expected to mediate LOCI’s effect on improved fidelity to digital MBC as experienced by youth (Aim 3). In this study, the clinic level is synonymous with the organization level; however, this is not always the case in applications of LOCI. The LOCI strategy can be applied to organizations with multiple levels, resulting in theoretical models that describe how LOCI intervenes at multiple organizational levels to influence climate

Figure 1 shows LOCI’s theoretical model as applied to MBC in the present study. This model forms the basis for the study hypotheses. The LOCI strategy draws on two leadership theories—full-range leadership [35, 36] and implementation leadership [37]—and on theories of organizational implementation climate [33, 34], to explain variation in implementation success and to identify targets for implementation improvement. As is shown in Fig. 1, LOCI seeks to equip first-level leaders (e.g., clinical program managers), with two types of leadership behaviors believed to influence implementation success. *Transformational leadership*, drawn from the full-range leadership model, is a general type of leadership that reflects a leader’s ability to inspire and motivate employees to follow an ideal or course of action [38, 39]. *Implementation* *leadership* is a type of focused leadership that refers to leader behaviors that facilitate the organization’s specific strategic objective of successfully implementing a focal EBP, such as MBC [37, 40–44]. As is shown in the figure, LOCI aims to increase first-level leaders’ use of these leadership behaviors in order to support the development of an implementation climate that prompts and supports clinicians’ use of the focal EBP with high fidelity.

Correlational studies offer preliminary support for the relationships shown in Fig. 1. In a 5-year study of 30 outpatient mental health clinics serving youth, Williams et al. [45] showed that increases in implementation leadership at the clinic level were associated with increases in clinic EBP implementation climate, which subsequently predicted increases in clinicians’ self-reported use of evidence-based psychotherapy techniques. Other studies have shown that higher levels of organizational implementation climate predict higher observed fidelity to evidence-based mental health interventions in outpatient clinics and schools [46, 47]. In the other fully powered trial of LOCI that is currently published [48], researchers studying mental health care in Norway showed that LOCI improved first-level leaders’ implementation leadership, transformational leadership, and clinic implementation climate for trauma-focused EBPs. However, no studies have tested the two key linkages in LOCI’s hypothesized theory of change, namely: (1) that improvement in clinic implementation leadership and transformational leadership contributes to subsequently improved clinic implementation climate, and (2) that improvement in clinic implementation climate explains LOCI’s effect on EBP fidelity.

### Study contributions

This study makes three contributions to the literature. In Aim 1, we test LOCI’s effects on growth in first-level leaders’ use of implementation leadership (Hypothesis 1) and transformational leadership (Hypothesis 2), and on clinic implementation climate for MBC (Hypothesis 3), from baseline to 18-month post-baseline (i.e., 6 months after completion of LOCI). This aim seeks to replicate findings from an earlier trial [48] in which LOCI improved these outcomes in a different treatment setting (mental health clinics within Norwegian health trusts), patient population (adults), and set of EBPs (trauma-focused assessment and psychotherapies). In Aim 2, we test the hypotheses that experimentally induced improvement in first-level leaders’ implementation leadership (Hypothesis 4) and transformational leadership (Hypothesis 5) at T_2_ (4 months after baseline) will mediate LOCI’s effects on clinic implementation climate at T_4_ (12 months after baseline). In Aim 3, we test LOCI’s focal mechanism, namely: that improvement in clinic implementation climate from pre- to post-LOCI (i.e., T_1_ to T_4_) will mediate LOCI’s effect on MBC fidelity during the same period (Hypothesis 6). We believe this is the first study to test whether experimentally induced improvement in clinic leadership contributes to improved implementation climate and whether improvement in implementation climate improves observed fidelity to an EBP.

## Method

### Study design and procedure

Project WISDOM was a cluster randomized, controlled, hybrid type III effectiveness-implementation trial designed to test the effects of LOCI versus training and technical assistance only on MBC fidelity in outpatient mental health clinics serving youth. Details of the trial and primary implementation and clinical outcomes are reported elsewhere [12]. The trial enrolled 21 clinics serving youth in Idaho, Oregon, and Nevada, USA. Clinics were eligible if they were not actively implementing a digital MBC system and if they employed three or more clinicians delivering psychotherapy to youth (ages 4–18 years). Using covariate constrained randomization, clinics were randomly assigned to one of two parallel arms: (1) LOCI plus training and technical assistance in MBC or (2) training and technical assistance in MBC only. Clinic-level randomization aligned with the scope of the LOCI strategy and prevented contamination of outcomes at the clinician and patient levels. Clinic leaders could not be naïve to condition; however, clinicians and caregivers of youth were naïve to condition.

Following baseline assessments and randomization of clinics, executives and first-level leaders in the LOCI condition began participating in the LOCI implementation strategy. One month later, clinicians who worked with youths in both conditions received training to implement an evidence-based digital MBC system called the Outcome Questionnaire-Analyst (OQ-A; see below for details; [49, 50]). Following the initial OQ-A training, clinics in both conditions received two booster trainings and ongoing OQ-A technical assistance from the OQ-A purveyor organization until the trial’s conclusion.

To assess LOCI’s effects on its targeted mechanisms of change, clinicians who served youth in participating clinics were asked to complete web-based assessments evaluating their clinic’s leadership and clinic implementation climate for MBC at five time points: baseline (T_1_; following randomization of clinics but prior to initiation of LOCI or OQ-A training), 4-month post-baseline (T_2_), 8-month post-baseline (T_3_), 12-month post-baseline (T_4_; coinciding with the conclusion of LOCI), and 18-month post-baseline (T_5_; 6 months after LOCI concluded). Surveys were administered from October 2019 to May 2021. Clinic leaders provided the research team with rosters and emails of all youth-serving clinicians at each time point. Confidential survey links were distributed by the research team directly to clinicians via email. Clinicians received a small financial incentive for completion of each assessment (i.e., gift card to a national retailer) based on an escalating structure (US $30, US $30, US $45, US $50, US $55).

The primary implementation outcome of MBC fidelity was assessed for new youth outpatients who initiated treatment in the 12 months following clinician training in the MBC system. Upon intake to services, parents/caregivers of new youth patients were presented with study information requesting their consent for contact by the research team. Caregivers who agreed were contacted by research staff via telephone to complete screening, informed consent, and baseline measures (if eligible). After study entry, caregivers completed assessments reporting on the youth’s treatment participation (i.e., number of sessions) and symptoms monthly for 6 months following the youth’s baseline. Assessments were completed regardless of the youth’s continued participation in treatment, unless the caregiver formally withdrew (*n* = 7). Caregivers received a US $15 gift card to a national retailer for completion of each assessment. Enrollment and collection of follow-up data for youth occurred from January 2020 to July 2021. The CONSORT and Stari guidelines were used to report the results of this mediation analysis within the larger trial [51, 52].

### Participants

All licensed clinicians who worked with youth in participating clinics at each time point were eligible to participate in web-based surveys of clinic leadership and climate. This broad inclusion criterion ensured a full picture of clinic leadership and climate at each time point.

Inclusion criteria for youth were intentionally broad to reflect the trial’s pragmatic nature and the applicability of MBC to a wide range of mental health diagnoses. Eligible youth were new patients (i.e., no psychotherapy at the clinic in the prior 12 months), ages 4 to 17 years, who had been diagnosed by clinic staff with an Axis I DSM disorder deemed appropriate for outpatient treatment at the clinic; it was not required that youths be assigned to clinicians who completed surveys. Youths were excluded if they initiated treatment more than 7 days before the informed consent interview. Electronic informed consent was obtained from all participants. The Boise State University Institutional Review Board provided oversight for the trial (protocol no. 041‐SB19‐081) which was prospectively registered at ClinicalTrials.gov (identifier: NCT04096274).

### Clinical intervention: digital measurement-based care

The OQ-A is a digital MBC system shown to improve the effectiveness of mental health services in over a dozen clinical trials across four countries [49, 53]. OQ-A measures are sensitive to change upon weekly administration and designed to detect treatment progress regardless of treatment protocol, patient diagnosis, or clinician discipline [53]. In this study, clinicians had access to parent- and youth-report forms of the Youth Outcomes Questionnaire 30.2 [54, 55] and the Treatment Support Measure [56, 57]. Measures were completed by caregivers and/or youth electronically (via tablet or phone). Administration typically took 3–5 min. Measures were automatically scored by the OQ-A system, and feedback reports were generated within seconds. Feedback included a graph of change in the youth’s symptoms, critical items (e.g., feelings of aggression), and a color-coded alert, generated by an empirical algorithm, indicating whether the youth was making expected progress or was at risk of negative treatment outcome.

Clinicians were instructed to administer a youth symptom measure to the caregiver and/or youth at each session, review the feedback within 7 days of the session, and use the feedback to guide clinical decision-making. Clinicians were encouraged to discuss feedback with the caregiver and/or youth when they believed it was clinically appropriate and to administer a Treatment Support Measure if a youth was identified as high risk for negative outcome. Consistent with prior MBC trials, clinicians were not given specific guidance on how to respond to feedback; instead, they were advised to use their clinical skills in partnership with patients and clinical supervisors.

### Implementation strategies

#### OQ-A training and technical assistance

The initial, 6-h, OQ-A training provided to clinicians in both conditions was conducted in-person by the OQ-A purveyor organization. Training focused on the conceptual and psychometric foundations of the measures, the value of clinical feedback, clinical application of measures and feedback with youth and families, and technical usage of the system. Learning activities included didactics, in vivo modeling and behavioral rehearsal, exercises with sample feedback reports, and use of the system in “playground mode.” Two, live, virtual, 1-h booster trainings were offered to clinicians 3 and 5 months after the initial training. Professional continuing education hours were offered at no cost for all trainings to encourage participation. After the initial training, all clinics in both conditions received year-round technical assistance from the OQ-A purveyor organization. This included on-demand virtual training sessions, an online library of training videos, and a customer care representative to troubleshoot technical issues.

### Leadership and Organizational Change for Implementation (LOCI)

Details of the LOCI implementation strategy are available elsewhere [11, 12]. Briefly, LOCI was implemented in quarterly cycles over 12 months. During each cycle, (1) executives and first-level leaders within LOCI clinics attended monthly organizational strategy meetings to review data and to develop clinic-wide policies, procedures, and practices to support OQ-A implementation, and (2) first-level leaders attended leadership development trainings (5 days total) and participated in brief (~ 15 min) weekly coaching calls, designed to enhance their leadership skills. Once per month, individual coaching calls were replaced by group coaching calls with all other first-level leaders in the LOCI condition.

To support enrollment in the study, clinic leaders in the training and technical assistance only condition were offered access to four, professionally produced, web-based, general leadership seminars (1 h each). Seminars covered general leadership topics like giving effective feedback and leading change. The seminars were made available immediately after the OQ-A training.

### Measures

#### MBC fidelity

The primary outcome of MBC fidelity was measured at the youth level using an empirically validated MBC fidelity index [22, 58, 59]. The index was generated using electronic metadata from the OQ-A system combined with monthly caregiver reports of the number of sessions youths attended. Following prior research [22], scores were calculated as the product of two quantities: (a) the youth’s completion rate (i.e., number of measures administered relative to the number of sessions attended within the 6-month observation period) and (b) the youth’s viewing rate (i.e., the number of feedback reports viewed by the clinician relative to the number of measures administered). Note that this product is equivalent to the ratio of viewed feedback reports to total sessions; it represents an events/trials proportion. MBC fidelity index scores summarize the level of MBC fidelity experienced by each youth (range = 0–1) and have been shown to predict clinical improvement of youths receiving MBC [22, 58, 59]. Importantly, this index captures the administration and viewing components of MBC fidelity but does not indicate whether clinicians used the feedback to guide treatment.

### Implementation leadership

Clinicians assessed the extent to which their first-level leaders exhibited implementation leadership behaviors with regard to the OQ-A using the 12-item Implementation Leadership Scale (ILS) [40]. The ILS includes four subscales assessing the extent to which the first-level leader is *proactive*, *knowledgeable*, *supportive*, and *perseverant* about implementation. Responses were made on a 0 (*not at all*) to 4 (*very great extent*) scale. Total scores were calculated as the mean of all items. In prior research, scores on the ILS exhibited excellent internal consistency, convergent and discriminant validity [40, 47, 60, 61], and sensitivity to change [45].

### Transformational leadership

Clinicians assessed the extent to which their first-level leaders exhibited transformational leadership behaviors using the Multifactor Leadership Questionnaire (MLQ) [62, 63]. The MLQ is a widely used measure that has demonstrated excellent psychometric properties [64] and is associated with implementation climate for EBP as well as clinicians’ attitudes toward, and use of, EBPs [65–68]. Responses were made on a 5-point scale (“not at all” to “frequently, if not always”). Consistent with prior studies, we used the 20-item transformational leadership total score, calculated as the mean of four subscales: idealized influence, inspirational motivation, intellectual stimulation, and individual consideration.

### Clinic implementation climate

Clinicians’ perceptions of their clinics’ implementation climate for OQ-A were measured using the 18-item Implementation Climate Scale (ICS) [34]. The ICS includes six subscales assessing *focus*, *educational support*, *recognition*, *rewards*, *selection*, and *openness*. Responses were made on a 0 (*not at all*) to 4 (*a very great extent*) scale with the total score calculated as the mean of all items. Prior research provides evidence for the structural, convergent, and discriminant validity of scores on the ICS [27, 34, 69–72] as well as sensitivity to change [45].

### Data aggregation

Best practice guidelines [73–76] recommend clinician ratings of first-level leadership and clinic implementation climate be aggregated and analyzed at the clinic level. To justify aggregation, guidelines recommend that researchers test the level of inter-rater agreement among clinicians within each clinic to confirm there is evidence of shared experience. We used the *r*_wg(j)_ statistic [77] to assess inter-rater agreement among clinicians within each clinic. Across all clinics and all waves, average values of *r*_wg(j)_ were above the recommended cutoff of 0.7 [78, 79] for implementation leadership (*M* = 0.82, *SD* = 0.27), transformational leadership (*M* = 0.87, *SD* = 0.24), and clinic implementation climate (*M* = 0.94, *SD* = 0.10).

### Covariates

In order to increase statistical power and to address potential imbalance across clusters, we planned a priori to include covariates of state and clinic size (number of youths served in the prior year) in all analyses. In addition, in the mediation analyses described below, we included baseline values of the hypothesized mediator and outcome (when possible) to increase the plausibility of the no-unmeasured-confounding assumptions within the causal mediation approach [80, 81].

### Data analysis

All analyses used an intent-to-treat approach. To test LOCI’s effects on growth in first-level leaders’ implementation leadership (H1), transformational leadership (H2), and clinic implementation climate (H3) for Aim 1, we used three-level linear mixed-effects regression models [82, 83] with random effects addressing the nesting of repeated observations (level 1) within clinicians (level 2) within clinics (level 3). Separate models were estimated for each outcome. At level 1, observations of leadership and climate collected from clinicians at each time point were modeled using a piecewise growth function that captured differences in change from baseline to each time point across conditions [84]. Implementation condition and clinic covariates were entered at level 3. Models were estimated using the mixed command in Stata 17.0 [85] under full maximum likelihood estimation, which accounts for missing data on the outcomes, assuming data are missing at random. Effect sizes were calculated as the standardized mean difference in *change* (i.e., difference in differences) from baseline to each time point (i.e., Cohen’s *d*) using formulas by Feingold [86]. Cohen suggested values of *d* could be interpreted as small (0.2), medium (0.5), and large (0.8) [87].

Aim 2 tested the hypotheses that experimentally induced improvement in first-level leaders’ implementation leadership (H4), and transformational leadership (H5) by T_2_, would mediate LOCI’s effect on improvement in clinic implementation climate by T_4_. These mediation hypotheses were tested using the multilevel causal mediation approach by Imai et al. [81], implemented in the R “mediation” package [88]. To align our analytic approach with our theoretical model, we estimated a 2–2-1 mediation model in which the primary antecedent (LOCI) and mediator (clinic-level aggregate leadership scores) entered the model at level 2 (i.e., the clinic level), and the outcome (clinician ratings of implementation climate) entered at level 1, representing latent clinic means [89]. Separate models were estimated for each type of leadership because Imai’s approach does not accommodate simultaneous mediators [81]. The inclusion of baseline values for the mediator (i.e., leadership) and outcome (i.e., climate) in each model modified the interpretation of the effects so that they represented the effect of LOCI on *change* in leadership from T_1_ to T_2_ and of change in leadership on change in climate from T_1_ to T_4_. To stabilize the effect estimates, we set the number of analytic simulations for the direct and indirect effects to 10,000. These analyses produced estimates of LOCI’s indirect and direct effects on T_4_ implementation climate, as well as the proportion of LOCI’s total effect on implementation climate that was mediated by improvement in leadership (i.e., proportion mediated = *p*_*m*_). Indirect effects indicate the extent to which LOCI influenced T_4_ implementation climate through its effect on T_2_ leadership (i.e., mediation). Direct effects indicate the residual (remaining) effect of LOCI on T_4_ implementation climate that was *not* explained by change in T_2_ leadership. The *p*_*m*_ statistic is an effect size measure indicating *how much* of LOCI’s effect on implementation climate was explained by change in leadership.

Aim 3 tested the hypothesis that improvement in clinic implementation climate from T_1_ to T_4_ would mediate LOCI’s effect on MBC fidelity during the same time period (H6). The nested data structure was accommodated using a 2–2-1 model in which the primary antecedent (LOCI) and mediator (aggregate clinic-level T_4_ implementation climate scores) occurred at level 2 (i.e., clinic level) and the outcome (MBC fidelity) occurred at level 1 (i.e., youth level). Note that the inclusion of baseline values of clinic implementation climate in this model modified the interpretation of the effects so that they represent the effect of LOCI on *change* in climate from T_1_ to T_4_ and of change in climate on fidelity during the same time period. To address the events/trials nature of the MBC fidelity index, a generalized linear mixed-effects model with random clinic intercepts, a binomial response distribution, and a logit link function was used in the second step of the mediation analysis [82]. In total, 18 clinics enrolled a total of 234 youth, all of whom had MBC fidelity data; however, one clinic was missing ratings of T_4_ implementation climate, resulting in a sample of 17 clinics and 231 youth for this analysis. A sensitivity analysis based on mean imputation of the missing T_4_ implementation climate value yielded the same inferential conclusions. A priori statistical power analyses conducted with the PowerUp! macro [90, 91] indicated the trial had power of 0.74–0.90 to detect minimally meaningful mediation effect sizes depending on observed intraclass correlation coefficients and variance explained by covariates.

## Results

**Fig. 2:**
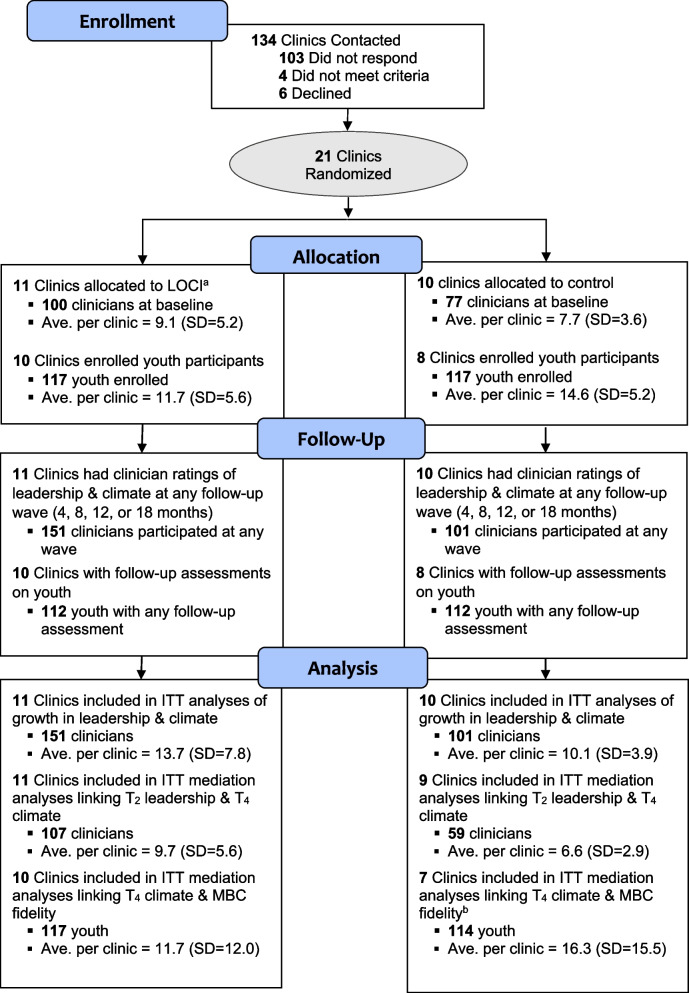
CONSORT diagram showing the flow of clinics, clinicians, and youth through the WISDOM trial Note: ITT, intent to treat; LOCI, Leadership and Organizational Change for Implementation strategy; *T*_2_, 4-month follow-up; *T*_4_, 12-month follow-up; WISDOM, Working to Implement and Sustain Digital Outcome Measures trial. ^a^One clinic participated in LOCI for only 6 months. ^b^One clinic that enrolled youth did not have T_4_ climate data

Figure 2 shows the flow of clinics, clinicians, and youth through the study. As is shown in Table 1, there were no differences by condition on the distribution of any clinic (*K* = 21), clinician (*N* = 252), or youth (*N* = 231) characteristics (all *p*s > 0.05). In total, 252 clinicians completed assessments for the study across 5 waves (average response rate = 88% across waves). The average number of participating clinicians per clinic was 12 (*SD* = 6.4). Nearly two-thirds of clinicians (*n* = 154, 61%) participated in 3 or more waves of data collection, and there were no differences by condition on clinician participation patterns (*p* = 0.114). A total of 234 youths were enrolled in the study. The average number of youths per clinic was 13.6 (*SD* = 13.3). The average number of assessments completed per youth was 5.9 (*SD* = 1.8) out of 7; 64% (*n* = 148) of youth had complete data, and 90% (*n* = 208) had 3 or more completed assessments. There were no differences in caregiver response rates for youth data by condition (*p* = 0.557).

**Table 1:** Characteristics of participating clinics, clinicians, and youth by condition

Characteristic	LOCI	Control	Total	*p*-value
**Clinic characteristic**				
No	11	10	21	
State, *n* (%)				0.926
Idaho	7 (63.6)	7 (70.0)	14 (66.7)	
Oregon	3 (27.3)	2 (20.0)	5 (23.8)	
Nevada	1 (9.1)	1 (10.0)	2 (9.5)	
Legal status, *n* (%)				0.465
Non-profit	5 (45.5)	3 (30.0)	8 (38.1)	
For-profit	6 (54.6)	7 (70.0)	13 (61.9)	
Implementing clinic-wide EBP at baseline, *n* (%)				0.653
Yes	5 (50.0)	6 (60.0)	11 (55.0)	
No	5 (50.0)	4 (40.0)	9 (45.0)	
FTE clinicians working with youth, *M* (SD)	10.1 (1.84)	8.0 (1.53)	9.1 (5.51)	0.393
Number of youths served (prior year), *M* (SD)	433.1 (89.9)	337.2 (52.6)	387.4 (343.5)	0.381
% revenue Medicaid, *M* (SD)	55.6 (8.21)	59.7 (9.52)	57.6 (27.4)	0.750
**Clinician characteristic**				
No	151	101	252	
Age in years, *M* (SD)	37.7 (0.8)	39.9 (1.1)	38.6 (0.7)	0.212
Years of clinical experience, *M* (SD)	5.46 (0.4)	6.37 (0.7)	5.83 (0.4)	0.339
Tenure at the organization, *M* (SD)	3.0 (0.3)	2.43 (0.3)	2.79 (0.2)	0.497
Exposure to MBC in graduate school, *M* (SD)	1.39 (0.1)	1.50 (0.1)	1.44 (0.1	0.406
Prior experience with MBC, *M* (SD)	1.53 (0.1)	1.71 (0.1)	1.6 (0.1)	0.307
Sex, *n* (%)				0.642
Male	26 (17.6)	19 (19.0)	45 (18.2)	
Female	119 (98.0)	78 (78.0)	197 (79.4)	
Prefer to self-describe	3 (2.0)	3 (3.0)	6 (2.4)	
Race, *n* (%)				0.060
American Indian or Alaskan Native	-	-	-	
Asian	4 (2.7)	1 (1.0)	5 (1.9)	
Black or African American	3 (2.0)	2 (2.0)	5 (1.9)	
Multiracial	2 (1.3)	-	2 (0.8)	
Pacific Islander or Hawaiian Native	2 (1.3)	-	2 (0.8)	
Prefer not to disclose	13 (8.6)	7 (7.0)	20 (7.9)	
Prefer to self-describe	9 (6.0)	4 (4.0)	13 (5.2)	
White	118 (78.2)	87 (86.1)	205 (81.4)	
Ethnicity, *n* (%)				0.236
Hispanic/Latinx	21 (14.3)	9 (9.0)	30 (12.2)	
**Child/family characteristic**				
No	117	114	231	
Child				
Age in years, *M* (SD)	11.3 (1.2)	12.1 (0.37)	11.7 (3.8)	0.501
Sex, *n* (%)				0.786
Male	54 (46.2)	52 (45.6)	106 (45.9)	
Female	62 (52.9)	62 (54.4)	124 (53.7)	
Prefer to not disclose	1 (0.9)	-	1 (0.4)	
Race, *n* (%)				0.373
American Indian or Alaskan Native	1 (0.9)	2 (1.8)	3 (1.3)	
Asian	1 (0.9)	1 (0.9)	2 (0.9)	
Black or African American	1 (0.9)	2 (1.8)	3 (1.3)	
Multiracial	10 (8.6)	6 (5.3)	16 (6.9)	
Pacific Islander or Hawaiian Native	-	-	-	
Prefer to self-describe	3 (2.6)	2 (1.8)	5 (2.2)	
White	98 (83.8)	100 (87.7)	198 (85.7)	
Ethnicity, *n* (%)				0.402
Hispanic/Latinx	21 (17.9)	14 (12.3)	35 (15.2)	
Received prior mental health services, *n* (%)	66 (56.4)	55 (48.3)	121 (52.4)	0.469
Family income, *n* (%)				0.965
Less than US $25,750	27 (23.1)	19 (16.7)	46 (19.9)	
US $25,751 to US $35,535	19 (16.2)	17 (14.9)	36 (15.6)	
US $35,536 to US $51,500	19 (16.2)	19 (16.7)	38 (16.5)	
More than US $51,500	52 (44.4)	59 (51.8)	111 (48.1)	
Parent highest education level, *n* (%)				0.998
High school graduate/GED or less	24 (20.5)	22 (19.3)	46 (19.9)	
Associate’s degree or some college	48 (41.0)	39 (34.2)	87 (37.7)	
Bachelor’s degree	26 (22.2)	28 (24.6)	54 (23.4)	
Graduate degree	18 (15.4)	24 (21.1)	42 (18.2)	
Baseline SAC total problem score, *M* (SD)	37.1 (1.2)	33.9 (1.2)	35.6 (0.9)	0.212
Number of sessions, *M* (SD)	10 (0.7)	10.9 (0.9)	10.4 (0.7)	0.589

To accommodate the nesting of clinicians and youths within clinics as well as multiple testing, *p*-values were generated using a bootstrap alpha on the basis of 500 resamples with replacement. *GED*, general education degree; *LOCI*, Leadership and Organizational Change for Implementation strategy; *M*, mean; *SAC*, Shortform Assessment for Children; *SD*, standard deviation

### Effects of LOCI on growth in clinic leadership and implementation climate

**Fig. 3:**
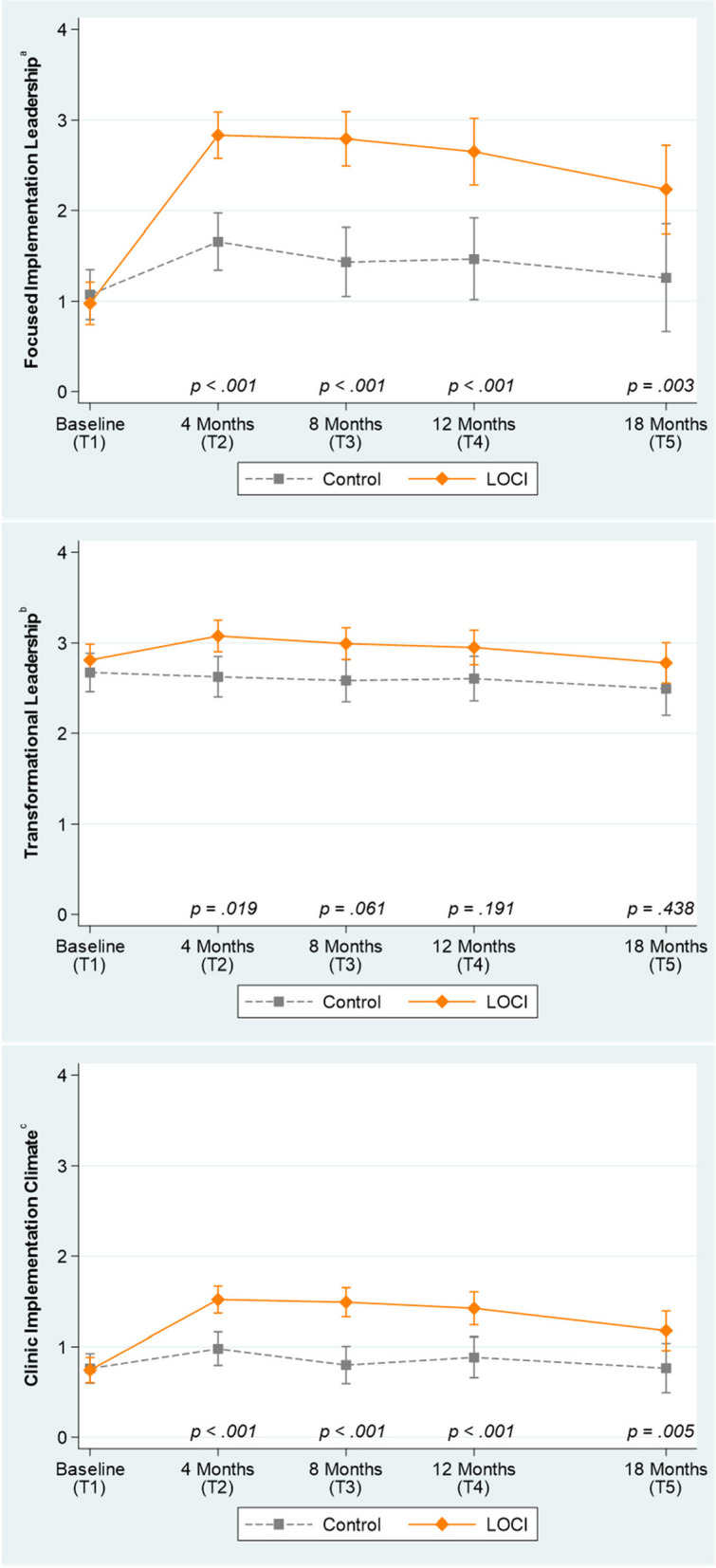
Change in clinic leadership and climate by condition and wave Note: Means estimated using linear mixed-effects regression models. Error bars represent 95% confidence intervals. All models control for state and clinic size. *P*-values contrast the difference between conditions on *change *in the outcome from baseline to the referenced time point. LOCI, Leadership and Organizational Change for Implementation condition. Control, training and technical assistance only condition. T5 occurred 6 months after completion of the LOCI strategy. See Table 2 for effect sizes. ^a^*K* = 21 clinics, *N* = 248 clinicians, *J* = 803 observations. ^b^*K* = 21 clinics, *N* = 251 clinicians, and *J* = 810 observations. ^c^*K* = 21 clinics, *N* = 247 clinicians, and *J* = 809 observations

Figure 3 shows the growth in first-level leaders’ implementation leadership, transformational leadership, and clinic implementation climate from baseline to 18 months (6 months after LOCI completed). Compared to clinicians in control clinics, clinicians in LOCI reported significantly greater increases in their first-level leaders’ use of implementation leadership behaviors from baseline to 4 months (*b* = 1.27, *SE* = 0.18, *p* < 0.001), 8 months (*b* = 1.46, *SE* = 0.22, *p* < 0.001), 12 months (*b* = 1.28, *SE* = 0.27, *p* < 0.001), and 18 months (*b* = 1.07, *SE* = 0.37, *p* = 0.003). These results supported Hypothesis 1. As is shown in Table 2, LOCI’s effects on implementation leadership were large at all follow-up points, including 4-, 8-, 12-, and 18-month post-baseline (range of *d* = 0.97 to 1.34).

**Table 2:** Effects of the Leadership and Organizational Change for Implementation (LOCI) strategy versus control on clinic leadership and clinic implementation climate by time

	Time 2 (4 months)		Time 3 (8 months)		Time 4 (12 months)		Time 5 (18 months)	
	*d*	95% *CI*	*d*	95% *CI*	*d*	95% *CI*	*d*	95% *CI*
Implementation leadership^a^ (*α* = 0.97)	**1.17**	0.85, 1.49	**1.34**	0.94, 1.73	**1.18**	0.69, 1.66	**0.97**	0.32, 1.62
Transformational leadership^b^ (*α* = 0.98)	**0.31**	0.05, 0.58	0.27	− 0.01, 0.55	0.21	− 0.10, 0.55	0.15	− 0.23, 0.52
Clinic implementation climate^c^ (*α* = 0.91)	**0.98**	0.63, 1.33	**1.25**	0.86, 1.63	**0.98**	0.55, 1.41	**0.76**	0.23, 1.28

Note: Reliability coefficients calculated as Cronbach’s alpha. Cohen *d* effect sizes express the standardized mean difference in *change* from baseline to the referenced time point, contrasting clinics assigned to LOCI versus control (training and technical assistance only). Effect sizes generated based on linear mixed-effect models and formulas by Feingold [86]; bolded values are statistically significant at *p* < .05. *CI* confidence interval

^a^*K* = 21 clinics, *N* = 248 clinicians, *J* = 803 observations

^b^*K* = 21 clinics, *N* = 251 clinicians, and *J* = 810 observations

^c^*K* = 21 clinics, *N* = 247 clinicians, and *J* = 809 observations

Hypothesis 2 stated that growth in first-level leaders’ transformational leadership would be superior in LOCI clinics relative to control. This hypothesis was partially supported (see Fig. 3 and Table 2). Clinicians in LOCI reported significantly greater growth in their first-level leaders’ use of transformational leadership behaviors from baseline to 4 months (*b* = 0.31, *SE* = 0.13, *p* = 0.019); however, this difference disappeared at 8 months (*b* = 0.27, *SE* = 0.14, *p* = 0.061) and was not evident at 12 months (*b* = 0.21, *SE* = 0.16, *p* = 0.191) or 18 months (*b* = 0.15, *SE* = 0.19, *p* = 0.438).

Hypothesis 3 stated growth in clinic implementation climate would be superior in LOCI clinics relative to control. This hypothesis was supported. Relative to clinicians in control, clinicians in LOCI reported significantly greater increases in their clinics’ implementation climate for MBC at 4 months (*b* = 0.56, *SE* = 0.10, *p* < 0.001), 8 months (*b* = 0.71, *SE* = 0.11, *p* < 0.001), 12 months (*b* = 0.55, *SE* = 0.12, *p* < 0.001), and 18 months (*b* = 0.43, *SE* = 0.15, *p* = 0.005). Table 2 shows that these effects were large during the intervention period and at posttest (i.e., at 4-, 8-, and 12-month post-baseline; *d* ranged from 0.98 to 1.25) and slightly attenuated at 18-month follow-up (*d* = 0.76).

### Indirect effects of LOCI on T_4_ implementation climate through T_2_ clinic leadership

Hypotheses 4 and 5 examined *how* LOCI improved T_4_ clinic implementation climate by testing mediation models. Hypothesis 4 stated LOCI would have an indirect effect on T_4_ implementation climate through improvement in first-level leaders’ use of implementation leadership from T_1_ to T_2_. As is shown in Table 3, this hypothesis was supported. The LOCI strategy had a significant indirect effect on T_4_ implementation climate through T_2_ implementation leadership (indirect effect = 0.51, *p* = 0.004) even as LOCI’s direct effect was not statistically significant (direct effect = 0.11, *p* = 0.582). The proportion-mediated statistic indicated 82% of LOCI’s total effect on clinic implementation climate at T_4_ was explained by improvement in implementation leadership from T_1_ to T_2_ (*p*_*m*_ = 0.82).

**Table 3:** Direct and indirect effects of the Leadership and Organizational Change for Implementation (LOCI) strategy on clinic implementation climate and digital MBC fidelity

LOCI effect	Outcome: T_4_ clinic implementation climate^a^								Outcome: MBC fidelity index^b^			
	Mediator: T_2_ implementation leadership				Mediator: T_2_ transformational leadership				Mediator: T_4_ clinic implementation climate			
		95% *CI*				95% *CI*				95% *CI*		
	Coeff	LL	UL	*p*	Coeff	LL	UL	*p*	Coeff	LL	UL	*p*
Indirect effect	**0.51**	0.15	0.95	.004	0.16	− 0.05	0.45	.135	**0.14**	0.01	0.37	.033
Direct effect	0.11	− 0.29	0.51	.582	**0.38**	0.05	0.72	.024	0.05	0.00	0.23	.482
Proportion mediated	**0.82**	0.25	1.00	.004	0.29	0.00	0.86	.136	**0.71**	0.02	1.00	.045

Effect estimates generated using Imai’s multilevel causal mediation R package [88]. Estimates in bold are statistically significant at *p* < .05 (two-tailed). All models include implementation condition (random assignment to LOCI vs. training and technical assistance only) as the focal antecedent plus covariates of state, clinic size, and baseline value of the mediator. *CI*, confidence interval; *MBC*, measurement-based care; *LL*, lower limit; *T*_2_, 4-month post-baseline; *T*_4_, 12-month post-baseline; *UL*, upper limit

^a^*K* = 20 clinics, *N* = 166 clinicians. These models also include baseline clinic implementation climate as a covariate. Indirect and direct effects indicate the change in T_4_ implementation climate (range = 0–4) caused by LOCI

^b^*K* = 17 clinics, *N* = 231 youth. Indirect and direct effects indicate the proportion difference in MBC fidelity (range = 0 − 1) caused by LOCI

Hypothesis 5 stated LOCI would have an indirect effect on T_4_ implementation climate through improvement in first-level leaders’ transformational leadership. This hypothesis was not supported (see Table 3). There was no evidence of an indirect effect of LOCI through transformational leadership (indirect effect = 0.16, *p* = 0.135) even as LOCI’s direct effect on T_4_ implementation climate remained statistically significant (direct effect = 0.38, *p* = 0.024). This pattern confirms LOCI improved T_4_ implementation climate but not through its effect on transformational leadership.

### Indirect effect of LOCI on MBC fidelity through implementation climate

Hypothesis 6 stated that LOCI’s effect on clinic implementation climate from T_1_ to T_4_ would mediate LOCI’s effect on MBC fidelity measured at the youth level during the same time period. As is shown in Table 3, results of the mediation analysis supported this hypothesis. The LOCI strategy had a statistically significant indirect effect on MBC fidelity through clinic implementation climate, increasing fidelity by 14 percentage points (indirect effect = 0.14, 95% *CI* = 0.01–0.37, *p* = 0.033) through this mechanism. The direct effect of LOCI on fidelity after accounting for clinic implementation climate was not statistically significant (direct effect = 0.05, *p* = 0.482). The proportion-mediated statistic indicated 71% of LOCI’s effect on MBC fidelity was explained by improvement in clinic implementation climate from T_1_ to T_4_ (*p*_*m*_ = 0.71, *p* = 0.045).

## Discussion

This study is the first to experimentally test the hypotheses that (a) increases in first-level leaders’ use of implementation leadership and transformational leadership improve clinic implementation climate, and (b) improvement in clinic implementation climate contributes to improved fidelity to an EBP. As such, it represents an important step in advancing recommendations for rigorous tests of mechanisms and causal theory in implementation science [73, 92]. Results support the hypotheses that (a) first-level leaders can help generate clinic implementation climates for a specific EBP through the use of implementation leadership behaviors, and (b) first-level leaders and organization executives can improve fidelity to EBP by developing focused implementation climates in their organizations.

In this trial, increases in first-level leaders’ use of implementation leadership by 4-month post-baseline explained 82% of LOCI’s effect on improvement in clinic implementation climate by 12-month post-baseline. This finding aligns with qualitative data from another recent trial of MBC implementation in community mental health [93], which also found that leader and clinical supervisor support for MBC were perceived as key implementation mechanisms. The linkage of implementation leadership to improvement in clinic implementation climate suggests implementation leadership behaviors are important targets for implementation success. Accordingly, pre-service educational programs for health leaders, implementation purveyor organizations, and other stakeholders interested in supporting EBP implementation should consider integrating these leadership competencies into core curricula and training.

Contrary to our hypotheses (see Fig. 1), LOCI did not exert lasting effects on transformational leadership, and increases in transformational leadership did not explain LOCI’s effect on improvement in clinic implementation climate. This pattern of results aligns with theoretical models of leadership and climate which suggest that specific types of focused leadership (i.e., implementation leadership [40]) are needed to generate specific types of focused organizational climate and associated outcomes [37, 44]. This finding also suggests the LOCI strategy could be streamlined without loss of efficacy by scaling back (or eliminating) components that address transformational leadership, as has been done in implementation studies of autism interventions [94]. Streamlining the content of LOCI may increase LOCI’s feasibility and allow for greater development of implementation leadership skills.

Improvement in clinic implementation climate explained 71% of LOCI’s effect on youth-level MBC fidelity. The validation of this theoretical linkage within an experimental design lends credence to prior correlational studies and theory suggesting clinic implementation climate can contribute to improved EBP implementation in mental health settings [27, 44–47]. These results suggest organizational and system leaders can improve the implementation of EBPs by deploying organizational policies, procedures, and practices that send clear signals to clinicians about the importance of EBP implementation relative to competing priorities within practice settings.

Discussions about change in organizational leadership and organizational climate often center around how long it takes for these constructs to change and what level of resources are required. Results from this trial suggest changes in first-level leadership and clinic implementation climate can occur quickly, within 4 months, and that these changes can be lasting, even 6 months after supports (i.e., LOCI) are removed. A similarly brief timeframe for initial change, and similarly sustained period of maintenance of effect, was observed for implementation leadership and climate in the other large trial of LOCI, which occurred in a different country, patient population, and EBP [48]. Together, results from these trials confirm implementation leadership and implementation climate are modifiable with a combination of training, weekly coaching calls, data feedback, and goal setting.

This study highlights multiple directions for future research. Future studies should examine moderators of LOCI’s effectiveness with an eye toward the minimally necessary components to make LOCI effective. For example, there is some evidence that intervention complexity moderates the association between implementation climate and fidelity [47]. This is consistent with organizational climate theory, which indicates climate is most strongly related to employee behaviors when service complexity is higher [95] and when there is high interdependence among employees to complete tasks and high intangibility of the service provided [96]. Other research should look beyond the organization level at how systems can be modified in complementary ways to create supportive implementation climates for targeted interventions.

Strengths of this study include the use of an experimental, longitudinal design, enrollment of clinics in diverse policy environments (i.e., three different States), measurement of leadership and climate by third-party informants whose behavior is most salient to implementation success (i.e., clinicians), measurement of MBC fidelity through objective computer-generated data, time ordering of hypothesized antecedents and consequents, and use of rigorous causal mediation models to estimate direct and indirect effects. The study’s primary limitation is generalizability given all clinics and caregivers of youth volunteered to participate. In addition, the MBC fidelity measure does not assess whether clinicians used the feedback to inform clinical decisions. Data were not collected on other mechanisms that may explain LOCI’s effects, a gap that may be fruitfully addressed by future qualitative research. Because the LOCI condition included training and technical assistance, it was not possible to isolate LOCI’s independent effects; this is also a fruitful area for future research. Finally, we were unable to fully test LOCI’s hypothesized theory of change due to our use of the causal mediation approach which precludes testing serial multiple mediator models (e.g., LOCI → leadership → climate → fidelity). Nonetheless, our results confirm the most consequential links in LOCI’s theoretical model and offer important directions for research and practice.

## Conclusion

In this mediation analysis of the WISDOM trial, experimentally induced improvement in implementation leadership explained increases in clinic implementation climate, which in turn explained LOCI’s effects on MBC fidelity in youth mental health services. This offers strong evidence that fidelity to EBPs can be improved by developing organizational leaders and strong implementation climates.

### Supplementary Information

**Additional file 1. **STARI checklist. CONSORT checklist.

## Data Availability

NJW and SCM had full access to all data in the study and take responsibility for the integrity of the data and the accuracy of the analyses. Requests for access to deidentified data can be sent to Dr. Williams at natewilliams@boisestate.edu, Boise State University School of Social Work, 1910 W University Dr., Boise, ID 83725.

## Abbreviations

DSM: *Diagnostic and Statistical Manual of Mental Disorders*
EBP: Evidence-based practice
H1–H6: Hypotheses 1–6
ILS: Implementation Leadership Scale
ICS: Implementation Climate Scale
LOCI: Leadership and Organizational Change for Implementation strategy
MBC: Measurement-based care
MLQ: Multifactor Leadership Questionnaire
OQ-A: Outcome Questionnaire-Analyst
WISDOM: Working to Implement and Sustain Digital Outcome Measures trial

## References

[1] Weiner BJ, Belden CM, Bergmire DM, Johnston M (2011). The meaning and measurement of implementation climate. Implement Sci.

[2] Damschroder LJ, Aron DC, Keith RE, Kirsh SR, Alexander JA, Lowery JC (2009). Fostering implementation of health services research findings into practice: a consolidated framework for advancing implementation science. Implementation Sci.

[3] Aarons GA, Hurlburt M, Horwitz SM (2011). Advancing a conceptual model of evidence-based practice implementation in public service sectors. Adm Policy Ment Health.

[4] Stetler CB, Ritchie JA, Rycroft-Malone J, Charns MP (2014). Leadership for evidence-based practice: strategic and functional behaviors for institutionalizing EBP. Worldviews Evid Based Nurs.

[5] Birken S, Clary A, Tabriz AA, Turner K, Meza R, Zizzi A, Larson M, Walker J, Charns M (2018). Middle managers’ role in implementing evidence-based practices in healthcare: a systematic review. Implement Sci.

[6] Nilsen P (2015). Making sense of implementation theories, models and frameworks. Implement Sci.

[7] Woodward J: Making things happen: a theory of causal explanation: Oxford university press; 2005.

[8] Meza RD, Triplett NS, Woodard GS, Martin P, Khairuzzaman AN, Jamora G, Dorsey S (2021). The relationship between first-level leadership and inner-context and implementation outcomes in behavioral health: a scoping review. Implement Sci.

[9] Williams NJ, Glisson C: Changing organizational social context to support evidence-based practice implementation: a conceptual and empirical review. In: Albers B, Shlonsky A, Mildon R, editors. Implementation Science 3.0. Switzerland: Springer; 2020, p. 145–172.

[10] Aarons GA, Ehrhart MG, Farahnak LR, Hurlburt MS (2015). Leadership and Organizational Change for Implementation (LOCI): a randomized mixed method pilot study of a leadership and organization development intervention for evidence-based practice implementation. Implement Sci.

[11] Aarons GA, Ehrhart MG, Moullin JC, Torres EM, Green AE (2017). Testing the Leadership and Organizational Change for Implementation (LOCI) intervention in substance abuse treatment: a cluster randomized trial study protocol. Implement Sci.

[12] Williams NJ, Marcus SC, Ehrhart MG, Sklar M, Esp S, Carandang K, Vega N, Gomes A, Brookman-Frazee L, Aarons GA: Randomized trial of an organizational implementation strategy to improve measurement-based care fidelity and youth outcomes in community mental health. Journal of the American Academy of Child and Adolescent Psychiatry; in press. 10.1016/j.jaac.2023.11.010 PMC11265517 38070868

[13] Lewis CC, Boyd M, Puspitasari A, Navarro E, Howard J, Kassab H, Hoffman M, Scott K, Lyon A, Douglas S (2019). Implementing measurement-based care in behavioral health: a **review**. JAMA Psychiat.

[14] de Jong K, Conijn JM, Gallagher RA, Reshetnikova AS, Heij M, Lutz MC (2021). Using progress feedback to improve outcomes and reduce drop-out, treatment duration, and deterioration: a** multilevel meta-analysis**. Clin Psychol Rev.

[15] Rognstad K, Wentzel-Larsen T, Neumer S-P, Kjøbli J (2023). A systematic review and meta-analysis of measurement feedback systems in treatment for common mental health disorders. Adm Policy Ment Health.

[16] Tam H, Ronan K (2017). The application of a feedback-informed approach in psychological service with youth: systematic **review and meta-analysis**. Clin Psychol Rev.

[17] Lambert MJ, Whipple JL, Hawkins EJ, Vermeersch DA, Nielsen SL, Smart DW (2003). Is it time for clinicians to routinely track patient outcome? **a meta-analysis**. Clin Psychol Sci Pract.

[18] Zhu M, Hong RH, Yang T, Yang X, Wang X, Liu J, Murphy JK, Michalak EE, Wang Z, Yatham LN (2021). The efficacy of measurement-based care for depressive disorders: systematic review and meta-analysis of randomized controlled trials. J Clin Psychiatry.

[19] Jensen-Doss A, Haimes EMB, Smith AM, Lyon AR, Lewis CC, Stanick CF, Hawley KM (2018). Monitoring treatment progress and providing feedback is viewed favorably but rarely used in practice. Adm Policy Ment Health.

[20] Gilbody SM, House AO, Sheldon TA (2002). Psychiatrists in the UK do not use outcomes measures: national **survey**. Br J Psychiatry.

[21] Patterson P, Matthey S, Baker M (2006). Using mental health outcome measures in everyday clinical practice. Australas Psychiatry.

[22] Bickman L, Douglas SR, De Andrade AR, Tomlinson M, Gleacher A, Olin S, Hoagwood K (2016). Implementing a measurement feedback system: a tale of two s**ites**. Adm Policy Ment Health.

[23] Garland AF, Kruse M, Aarons GA (2003). Clinicians and outcome measurement: what’**s the use?**. J Behav Health Serv Res.

[24] de Jong K, van Sluis P, Nugter MA, Heiser WJ, Spinhoven P (2012). Understanding the differential impact of outcome monitoring: therapist **variables that moderate feedback effects in a randomized clinical trial**. Psychother Res.

[25] Lyon AR, Lewis CC, Boyd MR, Hendrix E, Liu F (2016). Capabilities and characteristics of digital measurement feedback systems: results **from a comprehensive review**. Administration and Policy in Mental Health and Mental Health Services Research.

[26] Mellor-Clark J, Cross S, Macdonald J, Skjulsvik T (2016). Leading horses to water: lessons from a decade of helping psychological therapy services use routine outcome measurement to improve pract**ice**. Adm Policy Ment Health.

[27] Williams NJ, Ramirez NV, Esp S, Watts A, Marcus SC: Organization-level variation in therapists’ attitudes toward and use of measurement-based care. Administration and Policy in Mental Health and Mental Health Services Research 2022:1–16. 10.1007/s10488-022-01206-1 PMC9617767 35851928

[28] Gleacher AA, Olin SS, Nadeem E, Pollock M, Ringle V, Bickman L, Douglas S, Hoagwood K (2016). Implementing a measurement feedback system in community mental health clinics: a case study of multilevel barriers and facilitators. Adm Policy Ment Health.

[29] Marty D, Rapp C, McHugo G, Whitley R (2008). Factors influencing consumer outcome monitoring in implementation of evidence-based practices: results **from the National EBP Implementation Project**. Adm Policy Ment Health.

[30] Kotte A, Hill KA, Mah AC, Korathu-Larson PA, Au JR, Izmirian S, Keir SS, Nakamura BJ, Higa-McMillan CK (2016). Facilitators and barriers of implementing a measurement feedback system in public youth mental health. Adm Policy Ment Health.

[31] Aarons GA, Farahnak LR, Ehrhart MG: Leadership and strategic organizational climate to support evidence-based practice implementation. In: Dissemination and implementation of evidence-based practices in child and adolescent mental health. edn. New York, NY, US: Oxford University Press; 2014: 82–97.

[32] Klein KJ, Sorra JS (1996). The challenge of innovation implementation. Acad Manag Rev.

[33] Klein KJ, Conn AB, Sorra JS (2001). Implementing computerized technology: an organizational analysis. J Appl Psychol.

[34] Ehrhart MG, Aarons GA, Farahnak LR (2014). Assessing the organizational context for EBP implementation: the development and validity testing of the Implementation Climate Scale (ICS). Implement Sci.

[35] Avolio BJ, Bass BM, Jung DI (1999). Re-examining the components of transformational and transactional leadership using the multifactor leadership. J Occup Organ Psychol.

[36] Bass BM, Avolio BJ (1990). The implications of transactional and transformational leadership for individual, team, and organizational development. Res Organ Chang Dev.

[37] Aarons GA, Ehrhart MG, Farahnak LR, Sklar M (2014). Aligning leadership across systems and organizations to develop a strategic climate for evidence-based practice implementation. Annu Rev Public Health.

[38] Bass BM (1999). Two decades of research and development in transformational leadership. Eur J Work Organ Psy.

[39] Bass BM (1997). Does the transactional–transformational leadership paradigm transcend organizational and national boundaries?. Am Psychol.

[40] Aarons GA, Ehrhart MG, Farahnak LR (2014). The Implementation Leadership Scale **(ILS): development of a brief measure of unit level implementation leadership**. Implement Sci.

[41] Schneider B, Ehrhart MG, Macey WH (2013). Organizational climate and culture. Annu Rev Psychol.

[42] Schneider B, Ehrhart MG, Mayer DM, Saltz JL, Niles-Jolly K (2005). Understanding organization-customer links in service settings. Acad Manag J.

[43] Barling J, Loughlin C, Kelloway EK (2002). Development and test of a model linking safety-specific transformational leadership and occupational safety. J Appl Psychol.

[44] Ehrhart MG, Schneider B, Macey WH: Organizational climate and culture: an introduction to theory, research, and practice. New York, NY, US: Routledge/Taylor & Francis Group; 2014.

[45] Williams NJ, Wolk CB, Becker-Haimes EM, Beidas RS: Testing a theory of strategic implementation leadership, implementation climate, and clinicians’ use of evidence-based practice: a 5-year panel analysis. Implementation Science 2020, 15(1). 10.1186/s13012-020-0970-7 PMC7006179 32033575

[46] Williams NJ, Becker-Haimes EM, Schriger SH, Beidas RS (2022). Linking organizational climate for evidence-based practice implementation to observed clinician behavior in patient encounters: a lagged analysis. Implementation Science Communications.

[47] Williams NJ, Hugh ML, Cooney DJ, Worley JA, Locke J: Testing a theory of implementation leadership and climate across autism evidence-based interventions of varying complexity. Behavior Therapy 2022. 10.1016/j.beth.2022.03.001 PMC9395730 35987547

[48] Skar A-MS, Braathu N, Peters N, Bækkelund H, Endsjø M, Babaii A, Borge RH, Wentzel-Larsen T, Ehrhart MG, Sklar M: A stepped-wedge randomized trial investigating the effect of the Leadership and Organizational Change for Implementation (LOCI) intervention on implementation and transformational leadership, and implementation climate. BMC health services research 2022, 22(1):1–15. 10.1186/s12913-022-07539-9 PMC8895588 35246135

[49] Lambert MJ, Whipple JL, Kleinstäuber M (2018). Collecting and delivering progress feedback: a **meta-analysis of routine outcome monitoring**. Psychotherapy.

[50] Shimokawa K, Lambert MJ, Smart DW (2010). Enhancing treatment outcome of patients at risk of treatment failure: meta-analytic and mega-analytic review of a psychotherapy quality assurance system. J Consult Clin Psychol.

[51] Schulz KF, Altman DG, Moher D (2010). CONSORT 2010 statement: updated guidelines for reporting parallel group randomised trials. J Pharmacol Pharmacother.

[52] Pinnock H, Barwick M, Carpenter CR, Eldridge S, Grandes G, Griffiths CJ, Rycroft-Malone J, Meissner P, Murray E, Patel A: Standards for Reporting Implementation Studies (StaRI) statement. bmj 2017, 356. 10.1136/bmj.i6795 PMC5421438 28264797

[53] Lambert MJ (2012). Helping clinicians to use and learn from research-based systems: the OQ-analyst. Psychotherapy.

[54] Ridge NW, Warren JS, Burlingame GM, Wells MG, Tumblin KM (2009). Reliability and validity of the youth outcome questionnaire self-report. J Clin Psychol.

[55] Dunn TW, Burlingame GM, Walbridge M, Smith J, Crum MJ: Outcome assessment for children and adolescents: psychometric validation of the Youth Outcome Questionnaire 30.1 (Y‐OQ®‐30.1). Clinical Psychology & Psychotherapy: An International Journal of Theory & Practice 2005, 12(5):388–401.

[56] Harmon SC, Lambert MJ, Smart DM, Hawkins E, Nielsen SL, Slade K, Lutz W (2007). Enhancing outcome for potential treatment failures: therapist**–client feedback and clinical support tools**. Psychother Res.

[57] Whipple JL, Lambert MJ, Vermeersch DA, Smart DW, Nielsen SL, Hawkins EJ (2003). Improving the effects of psychotherapy: the **use of early identification of treatment and problem-solving strategies in routine practice**. J Couns Psychol.

[58] Bickman L, Kelley S, Breda C, De Andrade A, Riemer M (2011). Effects of routine feedback to clinicians on youth mental health outcomes: a **randomized cluster design**. Psychiatr Serv.

[59] Sale R, Bearman SK, Woo R, Baker N (2021). Introducing a measurement feedback system for youth mental health: predictors and impact of implementation in a community age**ncy**. Adm Policy Ment Health.

[60] Shuman CJ, Ehrhart MG, Torres EM, Veliz P, Kath LM, VanAntwerp K, Banaszak-Holl J, Titler MG, Aarons GA (2020). EBP implementation leadership of frontline nurse managers: validation of the implementation leadership scale in acute care. Worldviews Evid Based Nurs.

[61] Aarons GA, Ehrhart MG, Torres EM, Finn NK, Roesch SC (2016). Validation of the Implementation Leadership Scale **(ILS) in substance use disorder treatment organizations**. J Subst Abuse Treat.

[62] Bass BM, Avolio BJ: Manual for the multifactor leadership questionnaire (form 5X). Redwood City, CA: Mindgarden 2000.

[63] Avolio BJ: Full range leadership development: Sage Publications; 2010.

[64] Antonakis J, Avolio BJ, Sivasubramaniam N (2003). Context and leadership: an **examination of the nine-factor full-range leadership theory using the Multifactor Leadership Questionnaire**. Leadersh Q.

[65] Guerrero EG, Fenwick K, Kong Y (2017). Advancing theory development: exploring the leadership–climate relationship as a mechanism of the implementation of cultural competence. Implement Sci.

[66] Aarons GA (2006). Transformational and transactional leadership: association **with attitudes toward evidence-based practice**. Psychiatr Serv.

[67] Aarons GA, Sommerfeld DH (2012). Leadership, innovation climate, and attitudes toward evidence-based practice during a statewide implementation. J Am Acad Child Adolesc Psychiatry.

[68] Brimhall KC, Fenwick K, Farahnak LR, Hurlburt MS, Roesch SC, Aarons GA (2016). Leadership, organizational climate, and perceived burden of evidence-based practice in mental health services. Adm Policy Ment Health.

[69] Lyon AR, Cook CR, Brown EC, Locke J, Davis C, Ehrhart M, Aarons GA: Assessing organizational implementation context in the education sector: confirmatory factor analysis of measures of implementation leadership, climate, and citizenship. Implementation Science 2018, 13(1). 10.1186/s13012-017-0705-6 PMC5759223 29310673

[70] Williams NJ, Ehrhart MG, Aarons GA, Marcus SC, Beidas RS (2018). Linking molar organizational climate and strategic implementation climate to clinicians’ use of evidence-based psychotherapy techniques: cross-sectional and lagged analyses from a 2-year observational study. Implementation Sci.

[71] Ehrhart MG, Torres EM, Hwang J, Sklar M, Aarons GA (2019). Validation of the Implementation Climate Scale (ICS) in substance use disorder treatment organizations. Subst Abuse Treat Prev Policy.

[72] Ehrhart MG, Shuman CJ, Torres EM, Kath LM, Prentiss A, Butler E, Aarons GA (2021). Validation of the implementation climate scale in nursing. Worldviews Evid Based Nurs.

[73] Williams NJ (2016). Multilevel mechanisms of implementation strategies in mental health: integrating theory, research, and practice. Adm Policy Ment Health.

[74] Chan D (1998). Functional relations among constructs in the same content domain at different levels of analysis: a** typology of composition models**. J Appl Psychol.

[75] Lengnick-Hall R, Williams NJ, Ehrhart MG, Willging CE, Bunger AC, Beidas RS, Aarons GA (2000). Eight characteristics of rigorous multilevel implementation research: a step-by-step guide. Implementation Sci.

[76] LeBreton JM, Moeller AN, Wittmer JL (2023). Data aggregation in multilevel research: best practice recommendations and tools for moving forwar**d**. J Bus Psychol.

[77] James LR, Demaree RG, Wolf G (1984). Estimating within-group interrater reliability with and without response bias. J Appl Psychol.

[78] LeBreton JM, Senter JL (2008). Answers to 20 questions about interrater reliability and interrater agreement. Organ Res Methods.

[79] James LR, Choi CC (2008). Ko C-HE, McNeil PK, Minton MK, Wright MA, Kim K-i: Organizational and psychological climate: a **review of theory and research**. Eur J Work Organ.

[80] VanderWeele T: Explanation in causal inference: methods for mediation and interaction: Oxford University Press; 2015.

[81] Imai K, Keele L, Tingley D (2010). A general approach to causal mediation analysis. Psychol Methods.

[82] Raudenbush SW, Bryk AS: Hierarchical linear models: applications and data analysis methods, vol. 1: sage; 2002.

[83] Lang JW, Bliese PD, Adler AB (2019). Opening the black box: a** multilevel framework for studying group processes**. Adv Methods Pract Psychol Sci.

[84] Hedeker D, Gibbons R (2006). Longitudinal data analysi**s Johns Wiley & Sons**.

[85] StataCorp L: Stata statistical software: release 17 College Station. TX StataCorp LP 2021.

[86] Feingold A (2009). Effect sizes for growth-modeling analysis for controlled clinical trials in the same metric as for classical analysis. Psychol Methods.

[87] Cohen J: Statistical Power Analysis for the Behavioral Sciences, 2nd edn: Lawrence Erlbaum Associates; 1988.

[88] Imai K, Keele L, Tingley D, Yamamoto T: Causal mediation analysis using R. In: Advances in social science research using R: 2010: Springer; 2010: 129–154.

[89] Zhang Z, Zyphur MJ, Preacher KJ (2009). Testing multilevel mediation using hierarchical linear models: problems **and solutions**. Organ Res Methods.

[90] Kelcey B, Dong N, Spybrook J, Shen Z (2017). Experimental power for indirect effects in group-randomized studies with group-level mediators. Multivar Behav Res.

[91] Bulus M, Dong N, Kelcey B, Spybrook J: PowerUpR: power analysis tools for multilevel randomized treatments. R package version 1.1. 0. In.; 2021.

[92] Williams NJ, Beidas RS (2019). Annual Research Review: the state of implementation science in child psychology and psychiatry: a review and suggestions to advance the field. J Child Psychol Psychiatry.

[93] Lewis CC, Boyd MR, Marti CN, Albright K (2022). Mediators of measurement-based care implementation in community mental health settings: results from a mixed-methods evaluation. Implement Sci.

[94] Brookman-Frazee L, Stahmer AC: Effectiveness of a multi-level implementation strategy for ASD interventions: study protocol for two linked cluster randomized trials. Implement Sci 2018, 13(1). 10.1186/s13012-018-0757-2 PMC5944167 29743090

[95] Hofmann DA, Mark B (2006). An investigation of the relationship between safety climate and medication errors as well as other nurse and patient outcomes. Pers Psychol.

[96] Mayer DM, Ehrhart MG, Schneider B (2009). Service attribute boundary conditions of the service climate–customer satisfaction link. Acad Manag J.

